# PROTOCOL: Communication skills training for improving the communicative abilities of student social workers: A systematic review

**DOI:** 10.1002/cl2.1038

**Published:** 2019-09-05

**Authors:** Emma Reith‐Hall, Paul Montgomery

**Affiliations:** ^1^ Nottingham Trent University Nottingham United Kingdom; ^2^ University of Birmingham Birmingham United Kingdom

## 1 BACKGROUND

### 1.1 The problem

Good communication is central to social work practice (Koprowska, 2014; Lishman, 2009), underpinning the success of a wide range of social work activities. People in receipt of social work services value social workers who are warm, empathic, respectful, good at listening and demonstrate understanding (Beresford, Croft, & Adshead, 2008; Department of Health, 2002; Munford & Sanders, 2015; Social Care Institute for Excellence, 2000). Even in diverse and challenging circumstances, effective communication is thought to build constructive working relationships and enhance social work outcomes (Healy, 2018). Communication, sometimes referred to as interpersonal communication, is ‘a two‐way process. It involves two (or more) people interacting to exchange information and views’ (Beesley, Watts, & Harrison, 2018, p. 2). Hargie (2017) suggests interpersonal communication is driven and directed by the desire to achieve particular goals and is underpinned by perceptual, cognitive, affective and behavioural operations. In social work practice and education, the values of the profession and the speciﬁc social and cultural contexts in which social workers operate, influence the nature of interpersonal communication (Harms, 2015; Koprowska, 2014; Thompson, 2003).

Research has thus tended to focus on particular aspects of communication, or the impact of communication in specific contexts. In a study about how social workers communicate with parents in child protection scenarios, for example, it seems that social workers who demonstrated empathy towards simulated clients, encountered less resistance and more disclosure (Forrester, Kershaw, Moss, & Hughes, 2007). Social workers who use creative and play‐based approaches confidently, facilitate engagement and communication with children (Ferguson, 2016; Handley & Doyle, 2014). Adapting skills and strategies to address specific communication difficulties is equally important in social work with adults. Offering choices through pictographs (Rowland & McDonald, 2009) can help people with Aphasia to answer open questions, whilst Talking Mats can facilitate conversation with people who have dementia (Murphy, Gray, & Cox, 2007). Communicating effectively with adults in receipt of health and social care services enables them to better participate in important decisions about their care.

The impact of failing to communicate effectively has been well documented, particularly through reports into incidents of child deaths (Laming, 2003, 2009; Munro, 2011). Consequently, the importance of teaching communication skills to social work students to enable them to communicate effectively has long been recognised (Smith, 2002), followed by more recent calls for the expansion and/or improvement of this training (Luckock, Orr, Marchant, & Tanner, 2006; Narey, 2014). Considerable time, effort and money has been spent on achieving this aim, leading to a wide range of communication skills training courses becoming embedded in social work programmes across the globe. Communication generally, and some communication skills specifically, feature in the educational standards of different countries including the Australian Social Work Education and Accreditation standards, the Professional Capabilities Framework in the UK and the Educational Policy Accreditation Standards in the US (Australian Association of Social Workers, 2008; British Association of Social Workers, 2018, Council on Social Work Education, 2015).

### 1.2 The intervention

Communication skills training (CST) can be defined as ‘any form of structured didactic, e‐learning, and experiential (e.g., using simulation and role‐play) training used to develop communicative abilities’ (Papageorgiou, Loke, & Fromage, 2017, p. 6). Although ‘communication skills training’ (CST) is the name given to the intervention on a wide range of professional and vocational courses, in social work education, the intervention is more commonly referred to as the ‘teaching and learning of communication skills’; a trend reflected in the titles of various knowledge and practice reviews. Purpose, role and context have a significant impact on communication in social work practice, hence the need to integrate knowledge, values and skills has been recognised, with the conceptualisation of knowing, being and doing developed by Lefevre, Tanner, and Luckock (2008) becoming increasingly popular (Ayling, 2012; Woodcock Ross, 2016). In social work education, the intervention includes not only communication processes, but also an understanding of the broader contextual issues in which communication in social work practice occurs. This view of communication in social work as both an art and a science (Healy, 2018), alongside a move away from purely instructional methods, helps explain the preference for the term ‘teaching’ or ‘education’ rather than ‘training’ among social work academics and researchers. There is a tendency within this discipline for significant variation in terminology due to the wide knowledge base from which social work draws. The term ‘communication skills’ is not applied uniformly in the social work literature ‐ microskills, interpersonal skills and interviewing skills are frequently used alternatives.

In spite of a lack of consensus about what the intervention is called, Dinham (2006, p. 841) identified that ‘the inclusion of a dedicated communication skills module early in the course, or a strong communication component within an early module about methods, skills and practice’ is commonplace. A consensus appears to be emerging in the wider social work literature, in terms of what the basic communication skills for social work practice actually entail. These microskills, comprise non‐verbal communication such as making eye contact and nodding, alongside a range of verbal techniques including clarifying, reflecting, paraphrasing, summarising and asking open questions. Described in detail in a number of social work text books (Beesley, Watts and Harrison, 2018; Cournoyer, 2016; Healy, 2018; Sidell & Smiley, 2008), and featuring in the educational standards, competency and capability frameworks of various countries (Australian Association of Social Workers, 2008; British Association of Social Workers, 2018; Council on Social Work Education, 2015), these skills form part of the content of a number of communication skills courses and preparation for practice modules. Microskills help social workers and social work students to ‘establish and maintain empathy, communicate non‐verbally and verbally in effective ways, establish the context and purpose of the work, open an interview, actively listen, establish the story or the nature of the problem, ask questions, intervene and respond appropriately’ (Harms, 2015, p. 22). Microskills are considered to be transferable across client groups and settings.

Using case study scenarios that students might encounter in practice, microskills are rehearsed in relation to social work contexts. Typically, students practice the microskills through undertaking simulated social work tasks such as assessments and care planning. When applied to social work tasks and contexts, communication skills are sometimes referred to within the social work literature as interviewing skills. An interview is a 'person‐to‐person interaction that has a definite and deliberate purpose' (Kadushin & Kadushin, 2013, p. 6). It is through social work interviews that ‘important connections and relationships are developed, and where important concepts such as partnership and empowerment are taken forward’ (Trevithick, 2012, p. 185).

The pedagogic practices used to teach communication skills to social work students include a wide range of affective, cognitive and behavioural components, whereby students participate in a variety of activities. Following face‐to‐face taught input including theory, communication skills are generally rehearsed, using role‐play with peers (e.g., Koprowska, 2003), simulated practice with service users (e.g. Moss, Dunkerly, Price, Sullivan, Reynolds et al., 2007) or actors (e.g., Petracchi & Collins, 2006). Tutors and peers may also model communication skills to demonstrate different techniques. Critical reflection, which facilitates students' self‐awareness is encouraged. Feedback is an important component in helping learners develop an understanding of their strengths and areas for development, and a range of feedback mechanisms are welcomed by students (Tompsett, Henderson, Mathew Byrne, Gaskell Mew, & Tompsett, 2017). Video and playback are often used to support the learning that occurs through feedback and reflective processes. Some universities have purpose built video suites or provide students with recording equipment such as tablets to facilitate the recording of communication skills practice. The rationale for video and playback is ‘that each student's adult ability to be their own best assessor’ is ‘utilised to the full’ (Moss, Dunkerly, Price, Sullivan, Reynolds et al., 2007, p. 715); the value of which has been recognised by students elsewhere (Bolger, 2014; Cartney, 2006). A learning environment, characterised by trust, safety and security, appears to be an important mechanism for students to make use of experiential activities. Opportunities for observing skills in practice, through shadowing a social worker or allied practitioner, are a feature of some communication skills or preparation for practice modules. Attention may also be devoted to specific areas of communication: communicating with children, communicating with people who have hearing impairments, and inter‐professional communication are some examples.

No specific blueprint for communication skills training in social work exists, hence the nature of the training sessions and course length vary from one educational institution to another. Typically, communication skills training is delivered to first year undergraduate and postgraduate students before they commence their first practice placement: in England, this may comprise some of the 30 days of skills training which universities are required to provide. Content and teaching activities tend to be designed and delivered on an individual basis by social work academics, often with involvement from service users and carers, practitioners and local employers. Minimum requirements, dosage, and delivery methods are not prescribed, leading to considerable heterogeneity of the intervention. Subgrouping the different types of intervention and population, based on factors such as length of course and teaching methods, age and sex would be helpful, if a sufficient number of trials are found through the review.

### 1.3 How the intervention might work

Training or education‐based interventions aimed at improving the communicative abilities of student social workers seek to bring about changes in learners' knowledge, values and skills in terms of how to communicate effectively in social work practice.

Psychological perspectives and counselling theories, particularly humanistic and client‐centred theorists such as Rogers, Carkhuff and Egan tend to underpin microskills training. Other communication theories, including Hargie's (2006) model of interpersonal communication also provide a theoretical basis for the skills taught on some of these courses. Concerns have been raised that psychological and counselling theories have been applied to social work uncritically (Trevithick, Richards, Ruch, Moss, Lines et al., 2004), without due consideration of the challenges this may present. A number of social work academics have pulled together theory and research on communication skills in recent years (e.g., Beesley et al., 2018; Harms, 2015; Healy, 2018; Lishman, 2009; Woodcock Ross, 2016) in an attempt to address this issue. Nonetheless, it still remains ‘difficult to identify a coherent theoretical framework that informs the learning and teaching of communication skills in social work’ (Trevithick et al., 2004, p. 18).

The theoretical underpinnings of the pedagogic practices used to teach communication skills are not always clear (Dinham, 2006; Trevithick et al., 2004). Schön's (1983) conception of reflection in and on action and the importance of ‘learning by doing’ (1987, p. 17) are often cited as underpinning the teaching of communication skills modules in social work education. Experiential learning, ‘the process whereby knowledge is created through the transformation of experience’ (Kolb, 1984, p. 38) is another of the prevailing philosophies, although Trevithick et al. (2004, p. 24) suggest there is an uncritical assumption that ‘experiential is best’. Reference is sometimes made to theories of adult learning, whereby students are expected to draw on their own experiences, take responsibility for their own learning, and engage in peer learning. This mode of learning ‘is understood to encourage the sustained internalisation of skills' (Dinham, 2006, p. 847). Such ideas build on the concept of andragogy (Knowles, 1972, 1998) where mutual processes of learning and growth are encouraged.

The knowledge review conducted by Trevithick et al. (2004) identified articles where the theoretical foundation for teaching skills in social work were made explicit. The communication skills module at the University of York in the UK, based on Agazarian's theory, is located within a systems framework (Koprowska, 2003), whilst Edwards and Richards (2002) propose that relational teaching based on relational/cultural theory should underpin teaching in social work education, whereby mutual engagement, mutual empathy and mutual empowerment foster growth in relationships between tutors and students. These examples are the exception to the rule; few articles theorise the teaching and learning process (Eraut, 1994). Generally speaking, ‘communication skills have been taught, but not reflected upon; experienced, but not theorised’ (Moss, Dunkerly, Price, Sullivan, Reynolds et al., 2007, p. 711).

A wide variety of approaches for teaching communication skills to social work students exist in practice. Dinham (2006) suggests there is more expertise in the teaching and learning of communication skills than the literature denotes and encourages academics to continue theorising and researching this aspect of the curriculum. Although rigorous high quality evaluation of outcomes in social work education is still in the early stages of development (Carpenter, 2011), teaching communication skills to social work students is an aspect of the curriculum which has attracted considerable attention, therefore a review of the findings will help to clarify what we know about this important topic.

## 2 OBJECTIVES

This systematic review aims to critically evaluate all studies that have investigated the effectiveness of communication skills training programmes for social work students. The PICO (Population, Intervention, Comparator, Outcomes) framework and stakeholder collaboration informed the development of the research question. Student social workers constitute the population, communication skills training is the intervention under investigation, the absence of communication skills training or a course unrelated to communication are the comparators, and attitudes, knowledge, confidence and behavioural changes are the outcomes of interest. Stakeholders agreed that neither the comparator nor the outcomes should be specified within the research question itself, on the grounds that researchers and academics are unlikely to have specified these elements in the primary studies. The review builds on the work of Trevithick et al. (2004), but will not be restricted by the year of publication or language. The research question the review seeks to answer is, ‘What is the effectiveness of communication skills training for improving the communicative abilities of social work students?’ The review will seek to provide a robust evaluation of communication skills training for social work students and explain variations in practice to encourage evidence‐based teaching and learning in this important area of social work education.

### 2.1 Why it is important to do the review

A variety of communication skills courses have been proposed and are in practice in social work education. It is almost 15 years since a number of practice and knowledge reviews highlighted the lack of evaluation into communication skills courses, an issue which warranted further research (Trevithick et al., 2004; Dinham, 2006). To support this endeavour, methodological guidance for evaluating outcomes in social work education (Carpenter, 2005, 2011) has been produced. Consequently, a number of empirical studies (Koprowska, 2010; Lefevre, 2010; Tompsett, Henderson, Mathew Byrne, Gaskell Mew, & Tompsett, 2017) have sought to evaluate the teaching of communication skills among social work students, or investigate the impact of particular components of the intervention. Existing literature suggests that teaching social work students communication skills increases their self‐efficacy in terms of communicative abilities (Koprowska, 2010; Lefevre, 2010; Tompsett et al. 2017).

No comprehensive systematic review or meta‐analysis of this aspect of social work education has been undertaken and questions about whether or not teaching communication skills to social work students is effective, remain unanswered. It is time therefore to gather, summarise and synthesise empirical research so that the evidence can be used by educators and policy‐makers to guide decisions about which approaches are effective in teaching communication skills to social work students. In this time of political uncertainty and financial constraint, ‘it is important to accumulate evidence of the outcomes of social work education so that policy‐makers and the public can be confident that it is producing high‐quality social workers’ (Carpenter, 2016, p. 192), who are suitably equipped to deal with the demands of social work practice. We are conducting this systematic review to determine whether communication skills training for social work students works and which types of communication skills training, if any, are the most effective. To improve uptake and relevance, the systematic review is being developed in consultation with stakeholders (including academics, students, practitioners, service users and carers) and advice has been sought from leading social work organisations. It will further aim to point to areas where more research is required in this aspect of social work education.

## 3 METHODOLOGY

### 3.1 Criteria for including and excluding studies

#### 3.1.1 Types of study designs

All of the study designs must include an appropriate comparator to be eligible for inclusion in the review irrespective of whether outcome data are reported in a useable way. Studies designs can include: randomised trials, non‐randomised trials, controlled before‐after studies, interrupted time series studies and repeated measures studies. Interrupted time series studies that do not have a clearly defined point in time when the intervention occurred and at least three data points before and three after the intervention will be excluded. The justification for this wider range of study types is to identify any potential risk of harm which we hope to assess through wider evidence. Potential risk of harm would include any negative effects of CST on students' communicative abilities, for example, service users and carers might indicate that students' poor communication has left them feeling more confused, agitated, misunderstood or distressed (i.e., worse) than they did before the interaction.

To ensure quality of evaluation all studies will be critically appraised and an analysis of the results by study design will be considered. The comparison group will be composed of those who receive no educational intervention or those receiving educational interventions other than communication skills training. Trials comparing the effects of two different educational interventions to improve communication skills will also be included in this review. In accordance with Campbell policies and guidelines (The Campbell Collaboration, 2014), studies without comparison groups or appropriate counterfactual conditions will be excluded.

#### 3.1.2 Types of participants

All social work students taught communication skills on a generic qualifying social work course in a university setting will be included. This can include undergraduate and postgraduate students. Social work courses designed for a specific client group will be excluded, as will students on post‐qualifying courses.

#### 3.1.3 Types of interventions

Only studies in which the intervention group receives communication skills training and in which the control group receives nothing or receives an alternative training to the intervention group will be included. For the intervention, any underpinning theoretical model and any mode of teaching (taught input, videotape recording, role‐play with peers, simulated interviews with service users and carers) is considered acceptable. Interventions aimed at improving the communication skills of specific client groups for example children will be also be included in the review, providing they meet the inclusion criteria.

#### 3.1.4 Types of outcome measures

Outcomes will include changes in (a) knowledge, (b) attitudes, (c) confidence and (d) behaviours measured using objective and subjective scales. It is anticipated that these measures might be study‐speciﬁc rating scales, developed for use in evaluating communication skills. Stakeholder involvement indicated that behavioural change is an important outcome for all stakeholders. In addition, students and educators deemed confidence/self‐efficacy to be a relevant outcome. In keeping with the literature on outcomes in social work education (Carpenter, 2005, 2011), student satisfaction alone will not be considered as an outcome measure in the review.

#### 3.1.5 Types of settings

Interventions that take place in university settings will be included, however if a component of the intervention takes place in a practice setting, that will also be included and made clear in the analysis and report.

### 3.2 Search strategy

The search strategy will include multiple electronic databases, research registers, grey literature sources, and reference lists of reviews and relevant studies. Study selection will not be restricted by language, publication date or publication status.

To identify eligible studies the following data sources will be searched using the search strings set out in Appendix A:

a) Education Abstracts (EBSCO)
b) ERIC (EBSCO)
c) MEDLINE (OVID)
d) PsycINFO (OVID)
e) Web of Science/ Knowledge Database Social Science Citation Index
f) Social Services Abstracts (Proquest)
g) ASSIA Applied Social Sciences Index and Abstracts (Proquest)
h) ClinicalTrials.gov
  Relevant reviews will be searched for in the following databases:
i) Database of Abstracts of Reviews of Effectiveness
j) The Campbell Library
k) Cochrane Collaboration Library
l) Evidence for Policy Practice Information and Coordinating Centre (EPPI‐Centre)
  We will also search grey literature and the following databases and websites will be used:
m) Google Scholar – using a series of searches and screening the first 2 pages of results for each search
n) ProQuest Dissertations and Theses

We will search the reference lists of relevant trials and reviews in order to identify additional relevant studies. Web searches will be limited to the first 2 pages of search results. Prominent authors will be contacted for further information about their studies, and authors of included studies will be asked if they are aware of any other published or ongoing studies meeting our inclusion criteria. In addition, a final step towards the end of analysis, a manual search of the most recent issue(s) of key journals will be conducted. To do this, the top 5 journals that have provided included studies so far, will be identified and checked.

One reviewer will conduct the database searches, remove duplicates and irrelevant records. We anticipate that the searches will result in very few records to screen, so to ensure robustness, each record will be screened by title and abstract by two reviewers. Any studies deemed to be potentially eligible will be retrieved in full text and will be screened by two reviewers. Any disagreements will be discussed with colleagues until a consensus is reached.

The search strategy will be developed using the terms featuring in existing knowledge and practice reviews and in consultation with social work researchers and academics, to ensure that the broad range of terminology is included. Search strings will include terms relating to the intervention and population but not study design. Search strings and search limits will be modified for each database. Searching for exact phrases or proximity searching will be used to increase search specificity.

### 3.3 Description of methods used in primary research

Included studies will be any form of design where appropriate counterfactual conditions are satisfied, in accordance with the Cochrane Effective Practice and Organisation of Care guidelines for the inclusion of non‐randomised studies (Cochrane Effective Practice and Organisation of Care, 2017).

### 3.4 Criteria for determination of independent findings

To ensure that the effects of an individual intervention are only counted once, the following conventions will apply. Where there are multiple measures reported for the same outcome, an average effect size for each outcome will be calculated within each study. Where the same outcome construct is measured across multiple time domains, the main analysis will focus on synthesising the evidence relating to effect sizes at immediate post‐test. Any subsequent measures of outcomes beyond immediate post‐test will be meta‐analysed and reported separately.

### 3.5 Details of study coding categories

#### 3.5.1 Details of study coding categories

Once eligible studies have been found, an initial analysis of programme descriptions will be undertaken for all programmes. The Campbell data collection template form will be used to identify the core components of programmes and to develop an overarching typology and coding frame.

It is anticipated that such components are likely to include:

- Duration and intensity of the programme.
- Whether programme delivery included service users and carers
- Whether programmes used audio and video recording
- Whether communication skills were practised with peers, service users or actors
- Whether programmes included observation of social workers in practice
- The theoretical frameworks underpinning the intervention

Alongside extracting data on programme components, descriptive information for each of the studies will be extracted and coded to allow for sensitivity and subgroup analysis. This will include information regarding:

- study characteristics in relation to design, sample sizes, measures and attrition rates, and whether the study was conducted by a research team associated with the programme or an independent team.
- stage of programme development, for example whether it is a new programme being piloted or an established programme being replicated.
- participants' characteristics in relation to age, gender, ethnicity, geo‐political region and socio‐economic background.

Coding will be carried out by the review team independently and discrepancies will be discussed and a consensus agreed.

Quantitative data will be extracted to allow for calculation of effect sizes (such as mean change scores and standard error or pre and post means and standard deviations). Data will be extracted for the intervention and control group on the relevant outcomes measured in order to assess the intervention effects. If study reports do not contain sufficient data to allow calculation of effect size estimates authors will be contacted to obtain necessary summary data, such as means and standard deviations or standard errors.

Assessment of methodological quality and potential for bias will be conducted using The Cochrane Risk of Bias tool for randomised studies and the ROBINS‐I tool (2016) for non‐randomised studies. The overall quality of evidence relating to the primary outcomes will also be summarised using the new GRADE Guidance for Complex Interventions (unpublished).

### 3.6 Statistical procedures and conventions

#### 3.6.1 Approach to meta‐analysis

Random effects models, using inverse‐variance estimation, will be used as the basis for meta‐analysis, due to the likely heterogeneity of interventions that this review is likely to find. Analysis will be conducted using REVMAN. Heterogeneity will be measured and reported through the Q, I2 and Tau2 statistics.

#### 3.6.2 Calculation of effect sizes

It is anticipated that most outcomes reported will be based upon continuous variables and so the main effect size metric to be used for the purposes of the meta‐analyses will be the standardised mean difference, with a 95% confidence interval. Social work cohorts tend to be relatively small, so Hedges' g will be used to correct for any small sample bias. Where other effect sizes have been reported, such as Cohen's d or risk ratios (for dichotomous outcomes) these will be converted to Hedges' g for the purposes of the meta‐analysis. The formulae provided in the Cochrane Handbook (Higgins & Green, 2011) will be used for this purpose. Studies are likely to involve group‐level allocation, hence data will be included which have been adjusted to account for the effects of clustering, using multilevel modelling or adjusting estimates using the intra‐cluster correlation coefficient (ICC). If the effects of clustering have not been taken into account, estimates of effect size will be adjusted following guidance in the Cochrane Handbook.

### 3.7 Treatment of qualitative research

This systematic review is limited to synthesising the available evidence on the effectiveness of communication skills training to social work students. It is beyond the remit of this present review to synthesise the associated evidence related to process evaluations of such programmes hence we do not plan to include qualitative research.

## ROLES AND RESPONSIBILITIES

- Content: Emma Reith Hall
- Systematic review methods: Paul Montgomery
- Statistical analysis: Paul Montgomery
- Information retrieval: University of Birmingham library services
- Write up: Emma Reith Hall and Paul Montgomery

## SOURCES OF SUPPORT

Emma Reith Hall is hoping to undertake the systematic review as part of her PhD research, for which she receives ESRC funding.

We are particularly grateful to our stakeholders – the students, practitioners, service users and carers, social work academics and social work organisations who gave their input into the development of this systematic review.

## DECLARATIONS OF INTEREST

Emma Reith Hall teaches communication skills to social work students and is therefore involved in the design and delivery of the intervention at her place at work. She has conducted some primary research, but it would not fulfil the criteria required to be included in this review.

Paul Montgomery‐ None known.

## PRELIMINARY TIMEFRAME

Approximate date for submission of the systematic review.

January 2021

Submission is expected one year after the publication of the protocol.

January 2021

Please note this should be no longer than two years after protocol approval. If the review is not submitted by then, the review area may be opened up for other authors.

## PLANS FOR UPDATING THE REVIEW

Reviews should include in the protocol specifications for how the review, once completed, will be updated. This should include, at a minimum, information on who will be responsible and the frequency with which updates can be expected.

PM and ERH would anticipate updating the review every 5 years.

## AUTHOR DECLARATION

### Authors' responsibilities

By completing this form, you accept responsibility for preparing, maintaining and updating the review in accordance with Campbell Collaboration policy. The Campbell Collaboration will provide as much support as possible to assist with the preparation of the review.

A draft review must be submitted to the relevant Coordinating Group within two years of protocol publication. If drafts are not submitted before the agreed deadlines, or if we are unable to contact you for an extended period, the relevant Coordinating Group has the right to de‐register the title or transfer the title to alternative authors. The Coordinating Group also has the right to de‐register or transfer the title if it does not meet the standards of the Coordinating Group and/or the Campbell Collaboration.

You accept responsibility for maintaining the review in light of new evidence, comments and criticisms, and other developments, and updating the review at least once every five years, or, if requested, transferring responsibility for maintaining the review to others as agreed with the Coordinating Group.

### Publication in the Campbell Library

The support of the Coordinating Group in preparing your review is conditional upon your agreement to publish the protocol, finished review, and subsequent updates in the Campbell Library. The Campbell Collaboration places no restrictions on publication of the findings of a Campbell systematic review in a more abbreviated form as a journal article either before or after the publication of the monograph version in Campbell Systematic Reviews. Some journals, however, have restrictions that preclude publication of findings that have been, or will be, reported elsewhere and authors considering publication in such a journal should be aware of possible conflict with publication of the monograph version in Campbell Systematic Reviews. Publication in a journal after publication or in press status in Campbell Systematic Reviews should acknowledge the Campbell version and include a citation to it. Note that systematic reviews published in Campbell Systematic Reviews and co‐registered with the Cochrane Collaboration may have additional requirements or restrictions for co‐publication. Review authors accept responsibility for meeting any co‐publication requirements.

## APPENDIX A

### Search string

1) Population Concept: social work students
  (“social work student*” OR “student social worker*”)
2) Intervention Concept: communication skills training

a) (Communicat* OR Interpersonal OR Interview*)
  Note: Interviewing skills has a very specific meaning in social work that relates to communication in a range of social work encounters
b) (train* OR educat* OR teach* OR learn* OR curricul*)

**Combined search string**

**Table:**

“social work student*”			**OR**			“student social worker*”)		
**AND**								
Communicat^*^		**OR**	Interpersonal			**OR**		Interview*
**AND**								
train*	**OR**	educat*	**OR**	teach*	**OR**	learn*	**OR**	curricul*
